# Characterizing Differences in Endolymphatic Hydrops Signatures Among Meniere’s Disease Patients with and Without Migraine

**DOI:** 10.3390/medsci14010029

**Published:** 2026-01-07

**Authors:** Yoshiyuki Sasano, Fumihiro Mochizuki, Yusuke Ito, Erin Williams, Izumi Koizuka, Michael E. Hoffer, Manabu Komori

**Affiliations:** 1 Department of Otolaryngology, University of Miami Miller School of Medicine, Miami, FL 33136, USA or yoshiyuki.sasano@marianna-u.ac.jp (Y.S.); erin.williams@med.miami.edu (E.W.); michael.hoffer@miami.edu (M.E.H.); 2 Department of Otolaryngology, St. Marianna University School of Medicine, Kawasaki 216-8511, Kanagawa, Japan; ito_yusuke0728@yahoo.co.jp (Y.I.); koizuka@marianna-u.ac.jp (I.K.); manabu.komori@marianna-u.ac.jp (M.K.)

**Keywords:** Meniere’s disease, migraine, endolymphatic hydrops, endolymphatic contrast-enhanced MRI, HYDROPS

## Abstract

**Background/Objectives**: Migraine is frequently comorbid with Meniere’s disease, which may complicate interpretation of inner ear imaging and clinical diagnosis. While endolymphatic hydrops has been studied in Meniere’s disease and vestibular migraine separately, comparative imaging data for Meniere’s disease patients with and without migraine remain limited. **Methods**: We retrospectively analyzed 78 patients with definite Meniere’s disease who underwent endolymphatic contrast-enhanced MRI (HYbriD of Reversed image of Positive endolymph signal and native image of positive perilymph signal; or “HYDROPS”). Patients were classified as Meniere’s disease only group *(n* = 56), or Meniere’s disease with migraine (*n* = 22). The degree of endolymphatic hydrops (negative, mild, or significant) was assessed separately in the inner ear, the cochlea, and the vestibule. **Results**: In Meniere’s disease group, the affected ear consistently showed higher rates of significant endolymphatic hydrops compared to the healthy ear across the inner ear, cochlea, and vestibule (*p* < 0.01). In contrast, Meniere’s disease with migraine group showed no significant interaural differences. Meniere’s disease with migraine group showed a significantly higher frequency of significant endolymphatic hydrops in the healthy cochlea (*p* < 0.01). Similar patterns were observed in the inner ear (*p* < 0.025) and vestibule (*p* = 0.05), although these differences did not reach statistical significance. Bilateral hydrops was significantly more frequent in Meniere’s disease with migraine group than in Meniere’s disease group among all regions investigated (*p* < 0.05). **Conclusions**: Meniere’s disease patients with migraine exhibit a distinct endolymphatic hydrops pattern, characterized by bilateral or symmetrical hydrops and involvement of the healthy ear. These findings suggest migraine-related mechanisms may contribute to endolymphatic hydrops, and bilateral endolymphatic hydrops on endolymphatic contrast-enhanced MRI in suspected Meniere’s disease cases should prompt consideration of comorbid migraine, in addition to bilateral Meniere’s disease or asymptomatic hydrops.

## 1. Introduction

Ménière’s disease (MD) is characterized by recurrent vertigo attacks accompanied by auditory symptoms such as hearing loss, tinnitus, and aural fullness. Vestibular migraine (VM) is also a common cause of recurrent vertigo, and the diagnostic distinction between these two conditions is often based on the criteria established by the Bárány Society [1,2]. A major difference is that VM requires migraine features in at least 50% of vertigo episodes. However, both disorders present with recurrent vertigo, and because the current VM criteria do not include specific auditory symptoms, distinguishing MD from VM and determining whether both conditions are present can sometimes be challenging.

Although VM is frequently discussed in the context of differential diagnosis, the present study does not focus on diagnosing VM. Instead, the study aimed to evaluate the influence of comorbid migraine on endolymphatic hydrops (EH) in patients with MD. This point is particularly important because migraine has been reported to coexist in up to 56% of patients with MD [3], indicating that the two conditions frequently occur together and thereby complicate the diagnostic process. In the present study, migraine was defined according to the International Classification of Headache Disorders, 3rd edition (ICHD-3) [4]. Patients were categorized as having MD with or without migraine based on these criteria, and VM was not specifically diagnosed as a separate clinical entity in this cohort.

Several objective methods, such as vestibular evoked myogenic potentials (VEMP), electrocochleography, and gadolinium-enhanced inner ear MRI, have been reported in patients with VM to evaluate the presence of EH [5,6,7]. Murofushi et al. reported that the cVEMP tuning property test was positive in only 14.2% of VM patients [5]. In contrast, Seo et al. found positive VEMP-based indicators of EH in 9 of 14 VM cases [6]. G.rkov et al. evaluated 19 patients with VM using both electrocochleography and HYDROPS imaging. EH was detected in 10 patients using electrocochleography, but only in 4 patients using contrast-enhanced MRI, demonstrating a discrepancy between these modalities [7]. These studies suggest that the presence and extent of EH may differ in migraine-related conditions, although the findings have been inconsistent. Furthermore, research focusing on migraine in general and evaluating the association between migraine and EH remains limited.

In this study, we used endolymphatic contrast-enhanced MRI (HYbriD of Reversed image of Positive endolymph signal and native image of positive perilymph signal: “HYDROPS”) [8] to evaluate EH in MD patients with and without migraine.

## 2. Materials and Methods

### 2.1. Subjects and Ethics Approval

We retrospectively analyzed patients (*n* = 78) who presented with recurrent episodes of rotatory vertigo at our dizziness outpatient clinic between October 2021 and March 2022, who were suspected to have MD. All patients underwent pure-tone audiometry (PTA), eye movement recordings using infrared video-Frenzel goggles, the Dizziness Handicap Inventory (DHI) questionnaire, and HYDROPS. PTA was calculated using the four-frequency average method ({250 Hz + 500 Hz + 1000 Hz + 2000 Hz}/4).

The diagnosis of MD was based on the criteria proposed by the Bárány Society [1], and only patients classified as having “Definite MD” were included in this analysis. The diagnosis of comorbid migraine was made by a board-certified neurologist specializing in headache, based on the International Classification of Headache Disorders, 3rd edition (ICHD-3) [4]. Patients diagnosed with “Migraine without aura (1.1)” or “Migraine with aura (1.2)” were considered to have comorbid migraine. Patients with other primary headache disorders or secondary headache disorders were excluded. Migraine was diagnosed solely based on the ICHD-3 criteria, and otoscopic examination of the external auditory canal and tympanic membrane, as well as screening for inner ear inflammation or neoplastic lesions during HYDROPS imaging, was performed to differentiate migraine from headache attributed to disorders of the ear and to assess the presence of comorbidities.

Informed consent was obtained using an opt-out method in accordance with the protocol approved by the Institutional Review Board of St. Marianna University School of Medicine (Approval No. 5873). Individual written consent was waived due to the retrospective nature of the study.

### 2.2. HYDROPS

MRI scans were obtained using a 3.0 Tesla scanner with a 32-channel head coil. Intravenous gadolinium contrast was administered, and imaging was performed four hours post-injection using a heavily T2-weighted 3D-FLAIR sequence.

**Figure 1:**
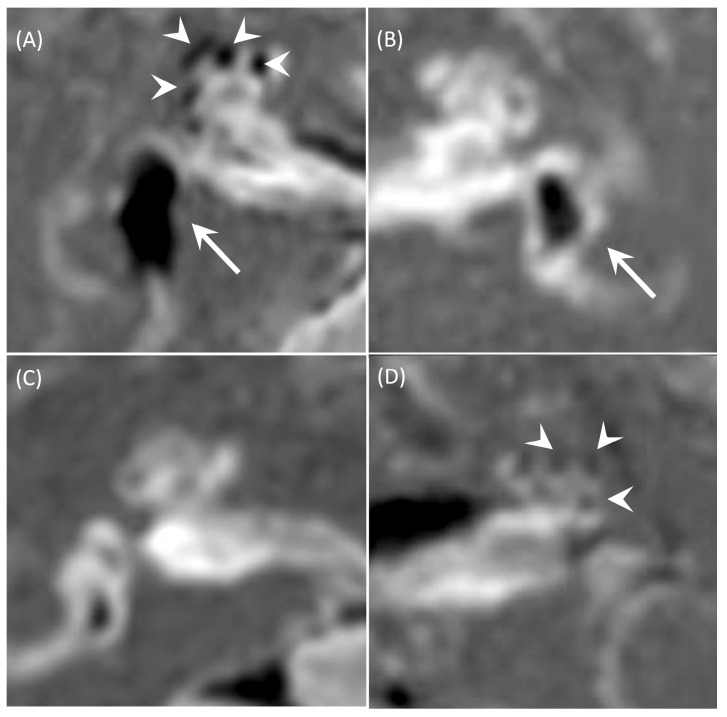
Representative HYDROPS images. (**A**) Significant vestibular and cochlear hydrops on the right ear. The arrow indicates the vestibule, and the arrowhead indicates the cochlea. (**B**) Mild vestibular hydrops, cochlear negative on the left ear. The arrow indicates the vestibule. (**C**) Vestibular and cochlear negative on the right ear. (**D**) Vestibular negative, mild cochlear hydrops on the left ear. The arrowhead indicates the cochlear.

EH was assessed using the criteria proposed by Nakashima et al. [9] (Figure 1). For the vestibule, endolymph occupying ≤33.3% of the total vestibular area was classified as negative, >33.3% and ≤50% as mild, and >50% as significant. For the cochlea, cases without displacement of Reissner’s membrane were classified as negative; cases in which the cochlear duct area was equal to or smaller than the scala vestibuli were classified as mild; and cases in which the cochlear duct area exceeded that of the scala vestibuli were classified as significant [9]. The degree of hydrops in the cochlea and vestibule was classified as “negative,” “mild,” or “significant” by consensus among three otolaryngologists specialized in vestibular disorders and two head and neck radiologists. Inner ear positivity was defined as a positive result in the vestibular and/or cochlear component. If findings of different degrees (significant, mild, or negative) coexisted, the strongest finding was used.

### 2.3. Statistical Analysis

Comparisons of age, age at onset, PTA, DHI scores, and disease duration between groups were performed using the Mann–Whitney U test. Fisher’s exact test was used for comparisons of sex and the presence of spontaneous nystagmus. All tests were two-tailed, and a *p*-value < 0.05 was considered statistically significant. Chi-square tests were used to compare the distribution of EH (unilateral vs. bilateral) between the two groups, and to compare the severity of hydrops (significant, mild, or negative) among the three categories. Bonferroni correction was applied to correct for Type I error due to multiple comparisons, setting the significance threshold at *p* < 0.0167 (0.05 ÷ 3). *p* values that did not meet this corrected threshold were considered statistically non-significant and were reported descriptively. All statistical analyses were performed using SPSS Statistics version 28.0 (IBM Corp., Armonk, NY, USA).

## 3. Results

### 3.1. Characteristics

**Figure 2:**
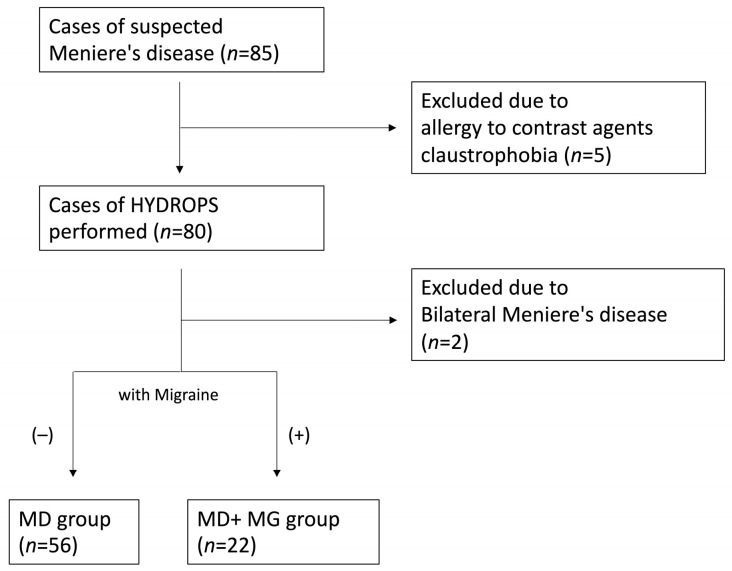
Diagnostic chart.

Five patients were excluded due to contraindications to contrast MRI, such as gadolinium allergy or claustrophobia. Additionally, two cases diagnosed with bilateral MD, showing bilateral sensorineural hearing loss, were excluded from this analysis. Fifty-six patients were assigned to the MD group, and 22 patients with MD and comorbid migraine were classified into the MD+Mg group (Figure 2).

Table 1 summarizes patient demographics and clinical characteristics for both the MD and MD+Mg groups, including age, sex, disease duration from onset of recurrent vertigo to HYDROPS imaging, age at onset, PTA in the affected and healthy ears, DHI scores, and peripheral eye movement disorder (PEMD) detected by infrared video-Frenzel goggles. Both age and age at onset were significantly lower in the MD+Mg group compared with the MD group (*p* = 0.01 and *p* = 0.03, respectively). No statistically significant differences were observed between the two groups in terms of sex, disease duration, PTA (affected and healthy ears), DHI scores, or PEMD.

### 3.2. EH in Each Group

The degree of EH in the inner ear of the affected and healthy ears in each group is presented in Table 2. In addition, the degree of EH in the vestibule and cochlea is shown separately in Table 3.

### 3.3. Comparison of EH Between Affected and Healthy Ears

#### 3.3.1. MD Group

**Figure 3:**
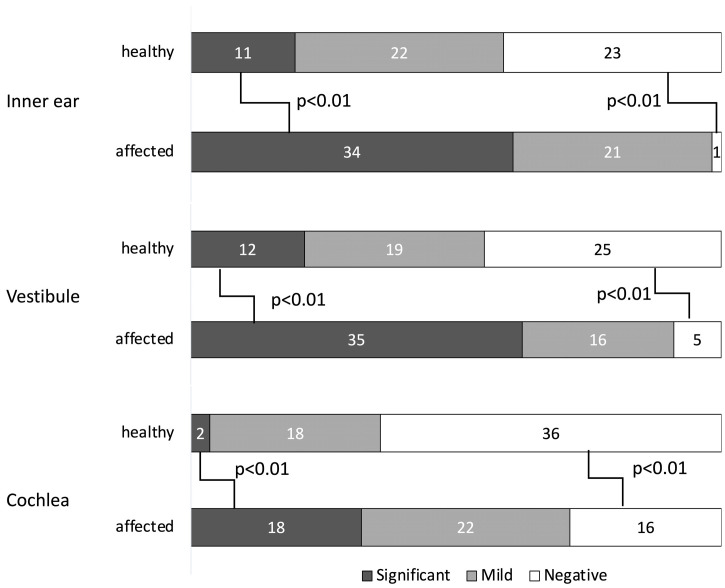
Degree of EH by region in the MD group. EH: Endolymphatic Hydrops; MD: Ménière’s disease.

Figure 3 shows the comparison of hydrops severity between affected and healthy ears in the MD group across the inner ear, vestibule, and cochlea.

Significant EH was more frequently observed in the affected ears in all regions (inner ear: *p* < 0.01; vestibule: *p* < 0.01; cochlea: *p* < 0.01), while negative cases were significantly fewer (all *p* < 0.01). No significant differences were observed in the number of mild hydrops cases (inner ear: *p* = 1.00; vestibule: *p* = 0.68; cochlea: *p* = 0.55). These findings indicate a significantly higher degree of hydrops in the affected ear compared to the healthy ear.

Significant EH was more frequently observed in the affected ears in all regions, while negative cases were significantly fewer compared with the healthy ears. There were no significant differences in the incidence of mild hydrops. Only pairwise comparisons with *p* ≤ 0.05 were shown in the figure.

#### 3.3.2. MD+Mg Group

**Figure 4:**
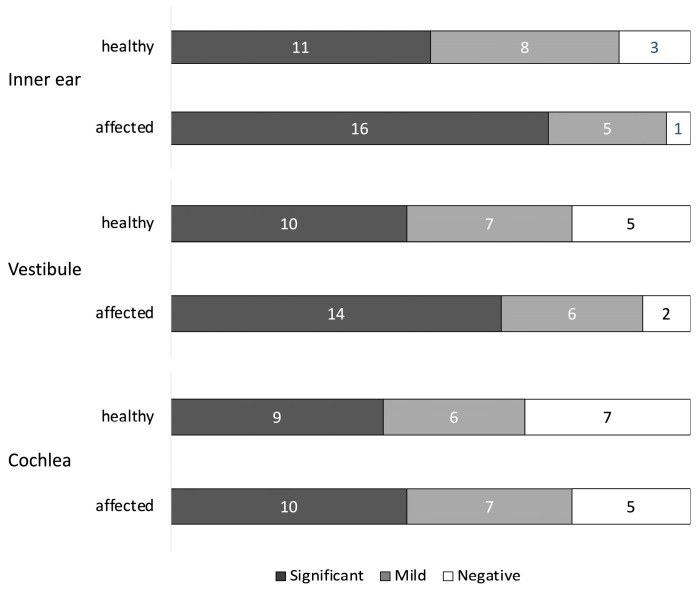
Degree of EH by region in the MD+Mg group. EH: Endolymphatic Hydrops; MD: Ménière’s disease; Mg: migraine.

Figure 4 shows the comparison of hydrops severity between affected and healthy ears in the MD+Mg group.

No significant differences were observed in any region between ears:

- Inner ear (significant: *p* = 0.22; mild: *p* = 0.51; negative: *p* = 0.61);
- Vestibule (significant: *p* = 0.36; mild: *p* = 1.00; negative: *p* = 0.41);
- Cochlea (significant: *p* = 1.00; mild: *p* = 1.00; negative: *p* = 0.74).

These results suggest no substantial difference in hydrops distribution between affected and healthy ears.

No significant differences were observed in any region. Only statistically significant pairwise comparisons are displayed in the figure.

### 3.4. Comparison of EH Between MD and MD+Mg Groups

The severity of EH at each site was compared between the two groups separately for the healthy and affected ear.

#### 3.4.1. Healthy Ear

**Figure 5:**
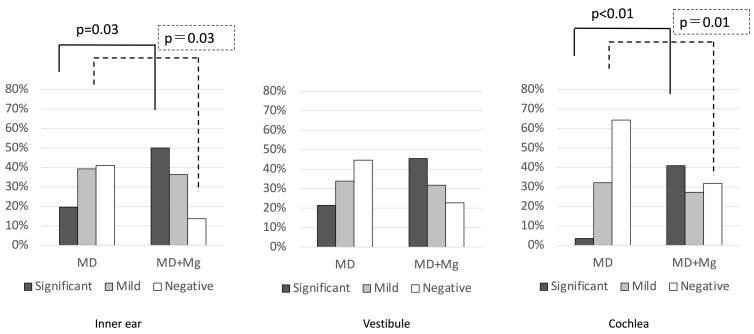
Comparison of EH in the unaffected ear between the two groups. EH: Endolymphatic Hydrops; MD: Ménière’s disease; Mg: migraine.

Hydrops severity in the inner ear, vestibule, and cochlea of the healthy ear was compared between the two groups (Figure 5). In the inner ear, a higher frequency of significant hydrops was observed in the MD+Mg group compared with the MD group; however, this difference did not reach statistical significance after Bonferroni correction (*p* = 0.03). No differences were observed in the frequency of mild hydrops (*p* = 1.00), and the proportion of negative cases was lower in the MD+Mg group, although this difference was not statistically significant (*p* = 0.03).

In the vestibule, significant hydrops were numerically more frequent in the MD+Mg group, but the difference was not statistically significant (*p* = 0.05). No differences were observed for mild or negative hydrops (*p* = 1.00 and *p* = 0.12, respectively).

In the cochlea, the MD+Mg group showed a significantly higher frequency of significant hydrops compared with the MD group (*p* < 0.01), while negative hydrops were significantly less frequent (*p* = 0.01). No significant difference was observed in the frequency of mild hydrops (*p* = 0.79).

Comparison of hydrops severity in the inner ear, vestibule, and cochlea of the healthy ear between the two groups. In the cochlea, the MD+Mg group showed a significantly higher frequency of significant hydrops compared with the MD group (*p* < 0.01), while negative hydrops were significantly less frequent (*p* = 0.01). Only pairwise comparisons with *p* < 0.05 were shown in the figure.

#### 3.4.2. Affected Ear

**Figure 6:**
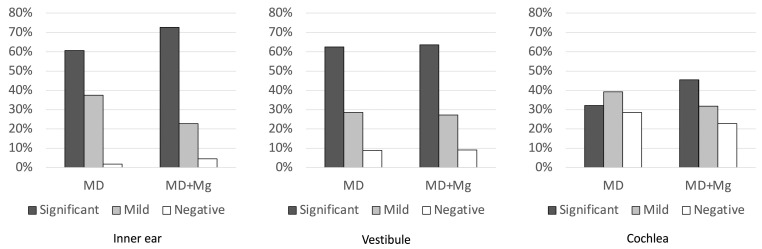
Comparison of EH in the affected ear between the two groups. EH: Endolymphatic Hydrops; MD: Ménière’s disease; Mg: migraine.

No significant differences were observed between the MD and MD+Mg groups in the affected ear in terms of hydrops severity in any region (Figure 6):

- Inner ear (significant: *p* = 0.43; mild: *p* = 0.29; negative: *p* = 0.49).
- Vestibule (significant: *p* = 1.00; mild: *p* = 1.00; negative: *p* = 1.00).
- Cochlea (significant: *p* = 0.30; mild: *p* = 0.61; negative: *p* = 0.78).

Hydrops severity in the affected ear showed no significant differences between the MD and MD+Mg groups in any region. Only statistically significant pairwise comparisons are displayed in the figure.

### 3.5. Laterality of EH in Positive Cases

Among cases with positive EH, we compared the laterality of EH between the two groups, categorizing cases as unilateral (hydrops limited to the affected ear) or bilateral (hydrops observed in both the affected and healthy ears).

**Figure 7:**
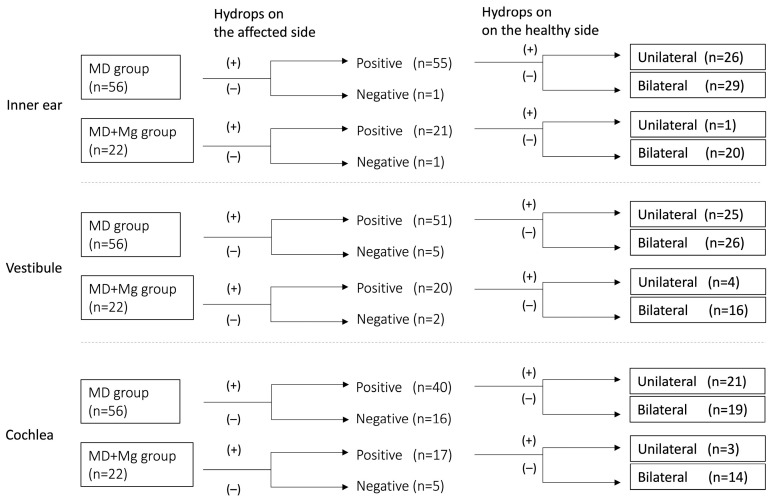
Number of unilateral and bilateral hydrops cases in each group. MD: Ménière’s disease; Mg: migraine.

The number of unilateral and bilateral cases in the inner ear, vestibular, and cochlear is shown in Figure 7.

**Figure 8:**
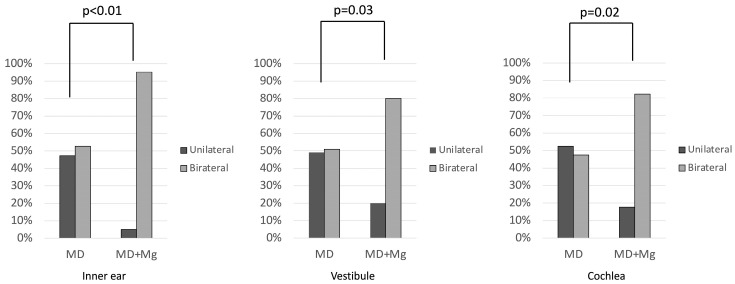
Comparison of unilateral and bilateral hydrops between groups by region. MD: Ménière’s disease; Mg: migraine.

Based on the results in Figure 7, a comparison was made between the two groups. Comparisons between the groups revealed that bilateral hydrops was significantly more frequent in the MD+Mg group than in the MD group in all regions (Figure 8).

The MD+Mg group showed more frequent bilateral hydrops across all regions.

## 4. Discussion

### 4.1. EH in MD Patients

Previous studies using the HYDROPS technique have reported a positive rate of EH in the affected ear of patients with MD ranging from 62.5% to 94.2% [10,11]. Regarding the healthy ear, Yoshida et al. [12] reported positivity rates of 46.9% in the vestibule and 53.2% in the cochlea, while Valerie et al. [13] reported 52.0% and 41.3%, respectively. In the present study, hydrops was detected in 98.2% of affected ears, 55.3% of vestibules, and 35.7% of cochleae in the healthy ear, results that are generally consistent with prior reports. Comparisons of the severity of hydrops between the affected and healthy ears revealed a significantly higher frequency of significant hydrops in the affected ear, which is in line with the findings of Yoshida et al. [12]. Even in the healthy ears of MD patients, positive hydrops findings were observed, though the severity was milder than in affected ears. The mechanism underlying hydrops formation in the healthy ear remains unclear, but it may reflect asymptomatic hydrops or subclinical disease progression as a manifestation of bilateral MD.

### 4.2. EH in MD with Comorbid Migraine

To the best of our knowledge, no prior studies have evaluated EH using the HYDROPS method specifically in MD patients with comorbid migraine. Reports involving cases of VM/MD-overlap syndrome (VM/MD-OS) are limited, with only Oh et al. [14] describing two hydrops-positive cases among eight patients, all showing positivity only in the affected ear (vestibule: 2 cases; cochlea: 1 case). Several studies have examined endolymphatic hydrops in VM using the HYDROPS technique. Nakada et al. [15] observed mild unilateral vestibular hydrops in one case, bilateral significant vestibular hydrops in another, and cochlear hydrops in five cases among seven patients with VM. Oh et al. [14] reported bilateral hydrops in 2 positive cases among 25 patients with VM. Valerie et al. [13] assessed 75 patients with VM and found hydrops positivity in the ipsilateral side in 23 inner ears, 15 cochleae, and 19 vestibules, and in the contralateral side in 17 inner ears, 12 cochleae, and 14 vestibules. These studies suggest a divergence in findings, with some showing low positivity rates in VM, while others report identifying a certain proportion of positive cases, indicating the lack of a unified conclusion.

In our study, three notable findings were observed regarding EH in MD patients with comorbid migraine: (1) No significant difference in hydrops severity (significant, mild, or negative) between the affected and healthy ears; (2) Although statistically significant differences were not observed in the inner ear and vestibule of the healthy ear after correction for multiple comparisons, the MD+Mg group consistently demonstrated numerically higher frequencies of significant hydrops compared with the MD group; (3) A significantly greater prevalence of bilateral hydrops compared to the MD group.

The relatively high prevalence of vestibular hydrops in the healthy ear observed in the MD+Mg group compared with previous reports may reflect a broader, bilateral inner ear involvement associated with migraine-related mechanisms rather than subclinical Ménière’s disease alone. These results suggest that in MD patients with comorbid migraine, hydrops in the healthy ear may not merely reflect subclinical MD or incidental findings, but rather that migraine itself may contribute to the formation of EH. Although significant differences were observed between the two groups in age at onset and age at examination, no significant difference was found in disease duration. This indicates that, while the timing of onset may vary between MD and MD+Mg patients, the overall length of the disease course is comparable. Therefore, the differences in MRI findings identified in this study are unlikely to be attributable to differences in disease stage, supporting the interpretation that migraine comorbidity itself may contribute to the observed variations in EH.

Although speculative, the possibility that migraine contributes to hydrops formation has been supported by a previous report in VM. Valerie et al. [13] proposed that repeated transient ischemia in the inner ear caused by migraine could lead to hydrops, resulting in MD-like symptoms. The trigeminovascular theory, a leading hypothesis in migraine pathophysiology, posits that neurogenic inflammation is mediated by neuropeptides such as calcitonin gene-related peptide (CGRP) released from trigeminal nerve endings, leading to central transmission of pain [16].

The trigeminal nerve provides sensory innervation to the cerebral and meningeal blood vessels, as well as to the vasculature of the inner ear via the anterior inferior cerebellar artery (AICA). It is known that the cochlear and vestibular labyrinth are also innervated by trigeminal fibers through orbital branches that affect parasympathetic regulation via the basilar artery and AICA [13]. Moreover, CGRP receptors are expressed in the vestibular nuclei, cochlear, and semicircular canals [16,17] and may mediate localized neuroinflammation, vascular permeability changes, vasodilation, and hydrops in these structures. Thus, while the involvement of migraine in hydrops formation remains hypothetical, the present findings suggest a potential link that warrants further investigation. Previous studies have indicated a higher prevalence of migraine in patients with bilateral MD [18,19]. Lopez et al. [18] suggested that patients with migraine may have a higher risk of developing MD in the second ear, although they emphasized that a cohort study would be required to confirm this. Migraine has also been reported to be common in patients with unilateral MD [1], and whether the EH observed in the healthy ear of the MD+Mg group in this study suggests that migraine may increase the risk of progression toward bilateral involvement remains unclear and requires further investigation. Our findings imply that when bilateral hydrops is detected in HYDROPS in patients suspected of having MD, clinicians should consider not only bilateral MD or asymptomatic hydrops in the healthy ear but also the possibility of comorbid migraine.

### 4.3. Limitations

Several limitations should be noted in this study. First, as this was a retrospective study, certain analyses were constrained by the available data. Specifically, in the MD+Mg group, we were unable to definitively classify all vertigo attacks as being attributable to MD or to migraine/VM, or to further subdivide attacks based on the presence or absence of cochlear symptoms. Similarly, comparisons among different types of migraine, such as migraine with aura versus migraine without aura, or chronic migraine versus episodic migraine, and evaluations of vertigo attack frequency were not feasible. Detailed information regarding the use of analgesics, including over-the-counter medications, was not consistently available, preventing analyses based on specific types or frequency of use. Nevertheless, no patients fulfilled the diagnostic criteria for typical medication-overuse headache. Because prior evidence regarding the expected magnitude of differences in EH between groups is limited, an a priori sample size calculation could not be performed. Given the variability of HYDROPS imaging and the heterogeneity of migraine characteristics, the statistical power may have been insufficient to detect subtle intergroup differences.

Another important limitation is the relatively small sample size, especially in the MD+Mg group. Due to this limited sample size, in this study, patients were categorized only into those with MD and those with MD+Mg, and a separate analysis of VM/MD-OS cases was not performed. This decision was based on our primary objective of evaluating the influence of migraine on hydrops formation in MD, as well as the concern that further subgrouping would substantially reduce the sample size and statistical power. The small sample size may also account for the discrepancy in sex distribution observed in this cohort, which differed from epidemiological data reported in Japan, where the male-to-female ratio is approximately 1:4 for migraine and 1:1.35 for MD [20,21]. Larger, prospective, multi-center cohorts will be required in future studies to specifically address VM/MD-OS cases and to validate the reproducibility and generalizability of our findings.

Finally, an additional limitation relates to the objective evaluation of EH using HYDROPS imaging. Particularly in 2D imaging, cochlear hydrops can be subtle and subject to interobserver variability, even when evaluated by consensus among three dizziness specialists and two radiologists specialized in otorhinolaryngology–head and neck imaging. Recent advances in three-dimensional volumetric analysis using HYDROPS have been proposed to address this issue [13], and such methods should be considered in future studies.

## 5. Conclusions

In this study, we applied the HYDROPS technique to patients with MD and comorbid migraine and found that the affected ear and the healthy ear frequently showed positive findings, with a higher prevalence of bilateral hydrops compared to MD-only patients. While further investigation is needed to clarify the pathophysiological relationship between migraine and EH, the presence of bilateral hydrops on MRI in suspected MD cases should prompt consideration of comorbid migraine, rather than being attributed solely to bilateral MD or asymptomatic hydrops in the healthy ear.

## Figures and Tables

**Table 1:** Comparison of subject characteristics between MD and MD+Mg groups.

	MD	MD+Mg	*p*
N	56	22	
Age (years), mean ± SD	52.5 ± 12.1	42.8 ± 17.7	0.01 *
Gender (F:M)	(25:31)	(11:11)	0.32
Disease duration (months), mean ± SD	61.3 ± 66.4	32.2 ± 25.8	0.14
Age at onset (years), mean ± SD	47.7 ± 13.0	40.1 ± 17.8	0.03 *
PTA affected side (dB), mean ± SD	38.1 ± 23.4	30.5 ± 27.0	0.17
PTA healthy side (dB), mean ± SD	16.2 ± 8.5	14.7 ± 6.5	0.59
DHI, mean ± SD	35.2 ± 24.9	39.1 ± 28.5	0.55
PEMD	(33/56)	(15/22)	0.31

An asterisk (*) indicates a statistically significant difference (*p* < 0.05). MD: Ménière’s disease; Mg: migraine; PTA: Pure-tone Audiometry; DHI: Dizziness Handicap Inventory.

**Table 2:** Degree of EH in affected and healthy ears in the inner ear for each group.

	Affected Ear				Healthy Ear			
	Positive	Significant	Mild	Negative	Positive	Significant	Mild	Negative
MD	55/56	34/56	21/56	1/56	33/56	11/56	22/56	23/56
	98.2%	60.7%	37.5%	1.8%	58.9%	19.6%	39.3%	42.1%
MD+Mg	21/22	16/22	5/22	1/22	19/22	11/22	8/22	3/22
	95.4%	72.7%	22.7%	4.6%	86.4%	50.0%	36.4%	13.6%

EH: Endolymphatic Hydrops; MD: Ménière’s disease; Mg: migraine.

**Table 3:** Degree of EH in affected and healthy ears in the vestibule and cochlea for each group.

Affected Ear								
	Vestibule				Cochlea			
	Positive	Significant	Mild	Negative	Positive	Significant	Mild	Negative
MD	51/56 (91.1%)	35/56 (62.5%)	16/56 (28.6%)	5/56 (8.9%)	40/56 (71.4%)	18/56 (32.1%)	22/56 (39.3%)	16/56 (28.6%)
MD+Mg	20/22 (90.9%)	14/22 (63.6%)	6/22 (27.3%)	2/22 (9.1%)	17/22 (77.2%)	10/22 (45.4%)	7/22 (31.8%)	5/22 (22.8%)
**Healthy Ear**								
	Vestibule				Cochlea			
	Positive	Significant	Mild	Negative	Positive	Significant	Mild	Negative
MD	31/56 (55.4%)	12/56 (21.4%)	19/56 (34.0%)	25/56 (44.6%)	20/56 (35.7%)	2/56 (3.6%)	18/56 (32.1%)	36/56 (64.3%)
MD+Mg	17/22 (77.2%)	10/22 (45.4%)	7/22 (31.8%)	5/22 (22.8%)	15/22 (68.2%)	9/22 (40.9%)	6/22 (27.3%)	7/22 (31.8%)

EH: Endolymphatic Hydrops; MD: Ménière’s disease; Mg: migraine.

## Data Availability

The datasets generated and analyzed during the current study are available from the corresponding author on reasonable request. These data include aggregated measurements, such as HYDROPS imaging evaluations, hearing test results, and patient demographic information. Individual patient identifiers and raw MRI data are not publicly available due to privacy and ethical restrictions.

## Abbreviations

The following abbreviations are used in this manuscript:

MD	Meniere’s Disease
VM	Vestibular Migraine
EH	Endolymphatic Hydrops
VEMP	Vestibular Evoked Myogenic Potentials
PTA	Pure-Tone Audiometry
HYDROPS	HYbriD of Reversed image of Positive endolymph signal and native image of positive Perilymph Signal
DHI	Dizziness Handicap Inventory
ICHD-3	International Classification of Headache Disorders, 3rd edition
PEMD	Peripheral Eye Movement Disorder
VM/MD-OS	Vestibular Migraine—Meniere’s Disease Overlapping Syndrome
CGRP	Calcitonin Gene-Related Peptide
AICA	Anterior Inferior Cerebellar Artery
